# Associations between individual hallucinogens and hallucinogen misuse among U.S. Adults who recently initiated hallucinogen use

**DOI:** 10.1016/j.abrep.2023.100513

**Published:** 2023-08-12

**Authors:** Grant Jones, Felipe Herrmann, Erica Wang

**Affiliations:** a Harvard University, United States; b University of Iowa, United States; c Colgate University

## Abstract

Hallucinogen dependence and abuse are DSM-IV diagnoses that are associated with significant morbidity, yet the specific hallucinogens that are most strongly linked to dependence and abuse are understudied. We used recent data from the National Survey on Drug Use and Health (2015–2020) and multivariable logistic regression to test the relationships that lifetime use of seven individual hallucinogens (MDMA/ecstasy, PCP, ketamine, psilocybin, LSD, peyote, and mescaline) shares with hallucinogen dependence and abuse among individuals who initiated hallucinogen use within the past two years (*N =* 5,252). We controlled for various demographic factors (sex, age, race/ethnicity, educational attainment, self-reported engagement in risky behavior, annual household income, marital status) and lifetime use of various substances. Lifetime PCP use was associated with increased odds of hallucinogen dependence or abuse (aOR [95% CI]: 6.27 [1.51, 26.0]). Additionally, PCP increased the odds of three main hallucinogen dependence and abuse criteria measures (aOR [95% CI]: 4.45 [1.11, 17.8], 5.58 [1.42, 22.0], and 7.01 [1.87, 26.3]). LSD conferred increased odds of two criteria (aOR: 2.33 [1.37, 3.98] and 2.53 [1.48, 4.33]), while ketamine and mescaline each conferred increased odds of one criterion (aOR: 2.12 [1.03, 4.39]; 5.39 [1.05, 27.7]). Future longitudinal studies and Bayesian statistical analyses can further assess the relationships between hallucinogens and disordered hallucinogen use.

## Highlights

• Hallucinogen dependence and abuse are debilitating but understudied DSM-IV diagnoses.
• We examined which hallucinogens were most strongly linked to these disorders.
• We used the National Survey on Drug Use and Health and logistic regression.
• PCP was most strongly linked to these conditions.

## 1 Introduction

Hallucinogen dependence and abuse are DSM-IV diagnoses and debilitating phenomena associated with a host of adverse psychiatric and behavioral outcomes such as lasting perceptual abnormalities, drug-induced psychosis, and physiological toxicity (Anthony et al., 1994, Johnson et al., 2018, Wu et al., 2008). “Hallucinogen abuse” is defined by the DSM–IV–TR as a “pattern of drug use marked by recurrent significant adverse consequences related to the repeated ingestion of hallucinogens”; “hallucinogen dependence” is defined as “a pattern of repeated or compulsive use of hallucinogens despite significant behavioral, physiological, and psychosocial problems associated with their use, as well as tolerance and characteristic withdrawal symptoms if use is suspended” (APA Dictionary of Psychology, 2007). Researchers estimate that 0.6–1.7% of the U.S. population suffers from hallucinogen dependence or abuse at some point in their lifetimes (Compton et al., 2005, Grant, 1996). However, hallucinogens are a heterogeneous class of substances including dissociative like ketamine and PCP (phencyclidine), naturally occurring classic psychedelics (psilocybin, lysergic acid diethylamide [LSD], mescaline), and the amphetamine-derivative MDMA/ecstasy. Furthermore, we are aware of very few comprehensive investigations into the individual hallucinogens that are most closely tied to dependence and abuse (Stone et al., 2007, Wu et al., 2010). The need to study which substances are most closely linked to hallucinogen dependence and abuse is particularly urgent as many hallucinogens are increasing in use in recreational and clinical contexts (Danforth et al., 2016, Livne et al., 2022, MacInnes et al., 2001, Tupper et al., 2015, Yockey et al., 2020). Thus, the investigation aims to explore the specific hallucinogens that have the strongest association with hallucinogen dependence and abuse in a population-based survey sample from the United States.

### 1.1 Dissociative Hallucinogens: Ketamine, PCP

A limited amount of research on PCP and ketamine has determined these substances to have significant potential for abuse and dependence. Conducted in 2010, one of the first longitudinal studies on individuals who use ketamine found increased ketamine use to be correlated with depression (C. J. Morgan et al., 2010); furthermore, the researchers from this study interpreted this finding as being driven by ketamine dependence resulting in commodity with depression, similar to the comorbidity with depression experienced by alcohol and opiate-dependent populations (C. J. Morgan et al., 2010, Palomo et al., 2007). Additionally, a 2001–2002 U.S. survey study found individuals with recent-onset use of PCP, but not ketamine, to be at excess risk of developing hallucinogen dependence (Stone et al., 2007). Given the limited amount of large-scale research directly exploring the abuse potential of PCP and ketamine, further investigation is needed to more deeply understand the potential that these substances have for abuse and dependence.

### 1.2 Classic Psychedelics: Psilocybin, LSD, Peyote, and mescaline

Classic psychedelics are naturally occurring substances that act as serotonin 2A receptor agonists and share similar psychoactive effects. These substances can cause distortions in one’s perception of time and space, create a sense of ego-dissolution (i.e., one’s sense of self has dissolved), and produce mystical-type experiences of lasting significance. Several comprehensive reviews and investigations have found these substances to have relatively low potential for chronic misuse (Bates and Trujillo, 2021, Johnson et al., 2018, Nichols, 2016). However, a 2007 study by Stone et al. found individuals with recent-onset use of mescaline to be at increased risk for developing hallucinogen dependence, while no such associations were found for individuals with recent-onset psilocybin, LSD, and peyote use (Stone et al., 2007). As this study stands in contrast to much of the evidence attesting to the low misuse potential of classic psychedelics, this finding calls for further investigation into the dependence and abuse potential of classic psychedelics that uses more recent data and aims to replicate these results.

### 1.3 Empathogens: MDMA/ecstasy

Many modern studies that observe hallucinogen dependence and abuse in recreational contexts have focused on MDMA/ecstasy. MDMA, commonly known by the street name “ecstasy” or “molly”, is a substance that can cause feelings of increased social connection, euphoria, and heightened energy levels (Montgomery and Roberts, 2022, ter Bogt et al., 2006, Wu et al., 2009). Stone et al. (2007) subsequently found an association between recent-onset use of MDMA/ecstasy and an excess risk of developing hallucinogen dependence in a nationally-representative sample of the U.S. population (Stone et al., 2007). Similarly, Wu et al. (2008) used a nationally-representative sample of the U.S. to demonstrate that one in five individuals who use MDMA/ecstasy reported at least one clinical feature of hallucinogen dependence and abuse (Wu et al., 2008). Additional follow-up work by Wu et al. (2009) found that one in three adolescents who use MDMA/ecstasy and nearly one in four adolescents who use other hallucinogens reported symptoms of hallucinogen dependence and abuse (Wu et al., 2009). Finally, Wu et al. (2010) conducted an item response theory analysis of individual DSM-IV criteria for hallucinogen abuse and dependence in adolescents and found individuals who use ecstasy to be at increased risk for dependence and abuse compared to individuals who use other hallucinogens. More recent investigations are needed to update and replicate previous correlational work showing an association between MDMA/ecstasy use and risk for dependence and abuse.

### 1.4 Current study

To address the gaps in the existing research on hallucinogen dependence and abuse, we use a similar methodological approach as Stone et al. (2007) to examine several individual hallucinogens and their potential for dependence and abuse in a large, diverse nationally representative sample of U.S. adults who have started using hallucinogens within the last two years (Stone et al., 2007). Our study can make an essential contribution to the scientific literature by attempting to replicate the results of Stone et al. (2007) and further assessing the dependence and abuse potential of various individual hallucinogens; to our knowledge, Stone et al. (2007) and Wu et al. (2010) represent some of the only studies to make such an assessment of hallucinogens thus far. While this approach cannot be used to determine causality, it can update and build upon preexisting work that explores the relationships between recent-onset use of specific hallucinogens and dependence and abuse.

## 2 Methods

Our study uses data from the National Survey on Drug Use and Health (NSDUH), an annual survey conducted to gain information on substance use and mental health in the United States. The NSDUH gathers information on U.S. citizens aged 12 and older. Additional information on the survey methodology is available at the NSDUH homepage (https://nsduhweb.rti.org/respweb/homepage.cfm). The current study uses six years of the latest NSDUH data (2015–2020) (United States Department of Health and Human Services. Substance Abuse and Mental Health Services Administration. Center for Behavioral Health Statistics and Quality., 2015, United States Department of Health and Human Services. Substance Abuse and Mental Health Services Administration. Center for Behavioral Health Statistics and Quality., 2016, United States Department of Health and Human Services. Substance Abuse and Mental Health Services Administration. Center for Behavioral Health Statistics and Quality., 2017, United States Department of Health and Human Services. Substance Abuse and Mental Health Services Administration. Center for Behavioral Health Statistics and Quality., 2018, United States Department of Health and Human Services. Substance Abuse and Mental Health Services Administration. Center for Behavioral Health Statistics and Quality., 2019, United States Department of Health and Human Services. Substance Abuse and Mental Health Services Administration. Center for Behavioral Health Statistics and Quality., 2020). In line with Stone et al., (2007), in our analyses we included adults aged 18 and older that had commenced hallucinogen use within two years of participating in the NSDUH survey (total unweighted *N =* 5,252). The study was exempt from IRB review as all data in this study are publicly available.

*Analyses.* We used survey-weighted multivariable logistic regression to assess the relationships that lifetime use of seven commonly used hallucinogens (MDMA/ecstasy, PCP, ketamine, psilocybin, LSD, peyote, and mescaline) shares with past year diagnosis of hallucinogen dependence or abuse, as well as the DSM-IV criteria for these conditions. We used the ‘Survey’ package in R version 4.1.2 to incorporate the complex survey design and sampling weights of the NSDUH into our analyses.

**Independent Variables and Covariates.** The independent variables in our study were binary variables (yes/no) that assessed lifetime use of seven commonly used hallucinogens: MDMA/ecstasy, PCP, ketamine, psilocybin, LSD, peyote, and mescaline. The following categorical demographic factors and binary (yes/no) substance use variables served as covariates in our analyses: sex (male or female), age (18–25, 26–34, 35–49, 50+), race/ethnicity (Non-Hispanic White, Non-Hispanic Black, Non-Hispanic Native American/Alaska Native, Non-Hispanic Native Hawaiian/Pacific Islander, Non-Hispanic Asian, Non-Hispanic more than one race, or Hispanic), educational attainment (5th grade or less, 6th grade, 7th grade, 8th grade, 9th grade, 10th grade, 11th grade, 11th or 12th grade, High School Diploma, Some College (No Degree), Associate’s Degree, College Degree or Higher), self-reported engagement in risky behavior (never, seldom, sometimes, or always), annual household income (less than $20,000, $20,000–$49,999, $50,000–$74,999, or $75,000 or more), marital status (married, divorced/separated, widowed, or never married), and lifetime use of various substances (inhalants, cocaine, heroin, tranquilizers, stimulants, sedatives, pain relievers, and marijuana).

**Dependent variables:** The main dependent variables for our study assessed past year diagnosis of hallucinogen dependence or abuse as well as the DSM-IV criteria for these conditions. The criteria are as follows:

Hallucinogen Dependence:

1. Spent a great deal of time over a period of a month or more getting, using, or getting over the effects of hallucinogens (*N* = 213)
2. Used hallucinogens more often than intended or was unable to keep set limits on hallucinogen use (*N* = 16)
3. Needed to use hallucinogens more than before to get desired effects or noticed that same amount of hallucinogen use had less effect than before (*N* = 216)
4. Inability to cut down or stop using hallucinogens every time one tried or wanted to (*N* = 15)
5. Continued to use hallucinogens even though they were causing problems with emotions, nerves, mental health, or physical problems (*N* = 76)
6. Hallucinogen use reduced or eliminated involvement or participation in important activities (*N* = 55)

Hallucinogen Abuse:

1. Serious problems at home, work, or school caused by using hallucinogens (*N* = 44)
2. Used hallucinogens regularly and then did something that might have put one in physical danger (*N* = 64)
3. Use of hallucinogens caused one to do things that repeatedly got one in trouble with the law (*N* = 15)
4. Problems with family or friends caused by using hallucinogens and continued to use hallucinogens even though you thought using hallucinogens caused these problems (*N* = 16)

To be classified as having hallucinogen dependence, participants were required to endorse at least three of the above criteria for hallucinogen dependence. To be classified as having hallucinogen abuse, one must not meet diagnostic criteria for dependence but endorse at least one of the above criteria for hallucinogen abuse. 433 participants in the NSDUH survey endorsed at least one of the above criteria for dependence or abuse.

Given the low rate of incidence of some of the criteria, we only included DSM-IV criteria as dependent variables if they had a frequency greater than *N* = 20, as a frequency lower than this number could result in models that yield spurious associations. Therefore, we ultimately included six main dependent variables in our study based on the aforementioned numbered DSM-IV criteria: criteria #1, #3, #5, #6, #7, and #8.

## 3 Results

### 3.1 Demographics

Table 1 details the demographics for individuals who do versus do not meet criteria for hallucinogen dependence or abuse, as well as the frequency of hallucinogen use by each of these two categories. We tested for demographic differences between these two categories using a chi-squared test with Rao & Scott's second-order correction. Individuals with hallucinogen dependence or abuse were more likely to report frequently engaging in risky behavior. There were no other demographic differences between the two groups.

**Table 1:** Demographics for individuals who do versus do not meet criteria for past year hallucinogen dependence or abuse.

Characteristic	Does not have hallucinogen dependence or abuse Unweighted *N* + (weighted %)	Has hallucinogen dependence or abuse Unweighted *N* + (weighted %)	p-value^1^
Marital Status			0.4
Married	345 (8.1%)	6 (7.4%)	
Widowed	27 (0.4%)	0 (0%)	
Divorced or Separated	158 (4.9%)	3 (0.5%)	
Never Been Married	4,581 (86%)	132 (92%)	
Age			0.2
18–25	4,333 (74%)	131 (83%)	
26–34	595 (19%)	7 (10%)	
35–49	157 (5.4%)	2 (1.5%)	
50+	26 (1.9%)	1 (5.2%)	
Sex			0.4
Male	2,830 (56%)	77 (61%)	
Female	2,281 (44%)	64 (39%)	
Race/ethnicity			0.7
Non-Hispanic White	3,094 (63%)	76 (58%)	
Non-Hispanic Black	481 (9.9%)	16 (13%)	
Non-Hispanic Native American/Alaska Native	81 (0.9%)	4 (1.2%)	
Non-Hispanic Native Hawaiian/Pacific Islander	22 (0.3%)	1 (0.2%)	
Non-Hispanic Asian	210 (5.4%)	9 (7.7%)	
Non-Hispanic more than one race	317 (3.6%)	7 (1.7%)	
Hispanic	906 (17%)	28 (18%)	
Yearly Household Income			0.3
<$20,000	1,617 (28%)	38 (23%)	
$20,000-$49,999	1,572 (30%)	49 (35%)	
$50,000-$74,999	652 (13%)	12 (7.3%)	
$75,000+	1,270 (30%)	42 (35%)	
Self-reported Engagement in Risky Behavior			<0.001
Never	971 (20%)	24 (16%)	
Seldom	2,137 (42%)	44 (26%)	
Sometimes	1,671 (33%)	49 (32%)	
Always	313 (5.9%)	23 (26%)	
Lifetime MDMA/Ecstasy Use	2,613 (51%)	89 (59%)	0.3
Lifetime PCP Use	58 (1.2%)	7 (14%)	<0.001
Lifetime Ketamine Use	232 (4.8%)	10 (11%)	0.14
Lifetime Peyote Use	96 (1.5%)	9 (4.4%)	0.067
Lifetime Mescaline Use	47 (1.4%)	5 (2.1%)	0.5
Lifetime Psilocybin Use	2,151 (41%)	60 (38%)	0.6
Lifetime LSD Use	2,343 (43%)	83 (54%)	0.079

1 chi-squared test with Rao & Scott's second-order correction.

### 3.2 Associations between individual hallucinogens and hallucinogen dependence and abuse

Table 2 provides the results from our models assessing the relationships between individual hallucinogens and overall hallucinogen dependence or abuse. Lifetime PCP use increased the odds of past year hallucinogen dependence or abuse (aOR [95% CI]: 6.27 [1.51, 26.0]). No other substances shared a relationship with hallucinogen dependence or abuse.

**Table 2:** Associations between lifetime use of individual hallucinogens and past year hallucinogen dependence or abuse + hallucinogen use frequency.

Lifetime Use	Frequency (unweighted *N*)	aOR (95% CI)^1^
MDMA/Ecstasy	2,702	1.50 (0.76, 2.95)
PCP	65	6.27* (1.51, 26.0)
Ketamine	242	1.12 (0.38, 3.32)
Peyote	105	1.73 (0.27, 11.0)
Mescaline	52	1.40 (0.27, 7.31)
Psilocybin	2,211	0.71 (0.41, 1.24)
LSD	2,426	1.45 (0.84, 2.50)

1 *p < 0.05; **p < 0.01; ***p < 0.001; aOR = adjusted odds ratio; CI = confidence interval.

Table 3 provides the results from our analyses that examine the associations between use of individual hallucinogens and DSM-IV criteria for hallucinogen dependence and abuse with frequencies greater than *N* = 20. PCP use was most regularly linked to dependence and abuse criteria and conferred increased odds of three outcomes: continuing to use despite emotional or physical health problems (aOR: 5.58 [1.42, 22.0]), engaging in fewer important activities as a result of use (aOR: 4.45 [1.11, 17.8]), and putting oneself in a physically hazardous situation following use (aOR: 7.01 [1.87, 26.3]). LSD conferred increased odds of two criteria: significant time spent getting, using, or getting over hallucinogens (aOR: 2.53 [1.48, 4.33]) and decreased effectiveness with continued use (aOR: 2.33 [1.37, 3.98]). Ketamine conferred increased odds of decreased effectiveness with continued use (aOR: 2.12 [1.03, 4.39]) while mescaline increased the odds of engaging in fewer important activities (aOR: 5.39 [1.05, 27.7]).

**Table 3:** Associations between individual hallucinogens and DSM-IV criteria for hallucinogen dependence and abuse (*N* > 20) (lifetime use of all other substances and all demographic factors included as covariates).

	Significant Time Spent Getting/Using	Decreased Effects/ Need More for Same Effect	Emotional/ Physical Health Problems	Fewer Important Activities	Significant Work/Home/School Problems	Use in Physically Hazardous Situations
Lifetime Use	aOR (95% CI)^1^	aOR (95% CI)	aOR (95% CI)	aOR (95% CI)	aOR (95% CI)	aOR (95% CI)
MDMA /Ecstasy	1.44 (0.92, 2.24)	1.37 (0.79, 2.37)	1.55 (0.68, 3.56)	3.13 (0.99, 9.86)	2.50 (0.81, 7.69)	1.32 (0.45, 3.84)
PCP	1.02 (0.33, 3.15)	1.28 (0.51, 3.22)	5.58* (1.42, 22.0)	4.45* (1.11, 17.8)	0.23 (0.03, 2.13)	7.01* (1.87, 26.3)
Ketamine	1.99 (0.91, 4.39)	2.12* (1.03, 4.39)	2.42 (0.96, 6.09)	0.74 (0.23, 2.42)	1.88 (0.38, 9.40)	0.64 (0.15, 2.66)
Psilocybin	0.99 (0.56, 1.76)	1.01 (0.61, 1.67)	1.01 (0.47, 2.17)	0.44 (0.15, 1.29)	0.71 (0.24, 2.05)	0.92 (0.42, 2.03)
LSD	2.53** (1.48, 4.33)	2.33** (1.37, 3.98)	1.50 (0.71, 3.17)	1.37 (0.60, 3.09)	1.65 (0.60, 4.54)	1.97 (0.71, 5.42)
Peyote	2.34 (0.88, 6.23)	0.97 (0.30, 3.10)	0.94 (0.15, 5.94)	1.42 (0.32, 6.21)	6.21 (0.75, 51.3)	2.04 (0.41, 10.1)
Mescaline	1.44 (0.39, 5.36)	1.48 (0.34, 6.37)	0.18 (0.01, 2.46)	5.39* (1.05, 27.7)	0.48 (0.02, 9.66)	2.52 (0.43, 14.6)

1 *p < 0.05; **p < 0.01; ***p < 0.001; aOR = adjusted odds ratio; CI = confidence interval.

## 4 Discussion

The goal of this study was to assess the associations that individual hallucinogens share with DSM-IV diagnoses of hallucinogen dependence and abuse among U.S. adults who recently initiated hallucinogen use. Our investigation within a large, representative sample of the U.S. population revealed PCP to be associated with hallucinogen dependence and abuse; additionally, among all hallucinogens in our study, it most regularly conferred increased odds of our main dependence and abuse criteria measures as well. LSD, ketamine, and mescaline also conferred increased odds of dependence and abuse criteria as well. MDMA/ecstasy and psilocybin did not share significant associations with hallucinogen dependence or abuse nor any of the criteria reported within this study. These findings provide critical insight into the link between hallucinogen use and dependence and abuse behavior and pave the way for causal investigations (e.g., longitudinal studies) into these associations.

### 4.1 Limitations

The main limitation to the present study is that it is based upon cross-sectional data, and therefore cannot be used to draw causal conclusions. Third variable factors (e.g., demographic differences) may underlie our observed findings. In addition, the data from the NSDUH are based on self-report measures. Downstream, self-reporting about sensitive information such as illicit substance use can result in the under-reporting of dependence and abuse, potentially skewing our results. Next, an additional limitation is that we did not explore frequency of use or dosage within this study, as the NSDUH has limited information on these factors for the hallucinogens included in this investigation. Future analyses that examine frequency and dosage can shed essential light on the relationships that individual hallucinogens share with dependence and abuse. Relatedly, this study also did not examine the effects of multiple substance use on dependence and abuse. Follow-up studies that examine how polysubstance use may increase the odds of hallucinogen misuse can also provide essential context for our findings. Lastly, an additional limitation is that we did not explore potential moderators and mediators of our observed associations. For instance, psychiatric comorbidities such as depression or anxiety may moderate or mediate the associations between individual hallucinogens and dependence and abuse behavior in our study. Future studies that control for comorbidity may provide even more information regarding the link between hallucinogens and dependence and abuse.

### 4.2 Mechanisms: Dissociatives (PCP, ketamine)

There are several potential mechanisms that may mediate the associations between PCP and ketamine use and dependence and abuse criteria.

First, hostile and violent behavior that results from PCP use may explain our findings linking this substance to increased odds of being in physically hazardous situations following hallucinogen use. PCP use is commonly associated with unpredictable hostile behavior such as intimate partner violence and general physical aggression (Bey and Patel, 2007, Crane et al., 2013). Furthermore, Bush (2013) noted that there was a 400% increase in emergency department visits between 2005 and 2011 that involved PCP, and this researcher hypothesized that this increase may be related to the PCP’s potential to elicit violence (Bush, 2013). Future research can clarify the potential relationship between PCP use, violence, and physical hazards that may result from using this substance.

Second, PCP use within club, rave, or party environments may be another underlying mechanism to explain the correlation between PCP use and increased odds of engaging in physically hazardous behaviors. Due to its dissociative and euphoria-inducing effects, PCP is often used at parties and in recreational settings (Ompad et al., 2004). Additionally, there is substantial evidence to suggest that party environments are associated with precarious behaviors, such as engaging in risky or excessive substance use, driving while intoxicated, and physical aggression (Duff and Rowland, 2006, Green and Plant, 2007, Palamar and Sönmez, 2022), all behaviors that would lead one to endorse the aforementioned DSM-IV criteria for hallucinogen abuse. Additional investigations are needed to assess how environmental factors may enhance the risk of PCP use.

Third, PCP’s long duration of intoxication may be a mechanism to explain the association between this substance and increased odds of engaging in fewer important activities. The acute effects of PCP use can last up to 48 h (Journey & Bentley, 2022), and effects of severe PCP intoxication can last up to six days (Bey & Patel, 2007). Additionally, some individuals who use PCP have been known to re-experience symptoms of intoxication days to months after initial use (Journey & Bentley, 2022). Further inquiries should explore the relationship between the length of PCP intoxication and potential reductions in engagement with meaningful activities.

Our findings linking ketamine to decreased effectiveness with continued use is consistent with the existing literature, which suggests that individuals develop a tolerance following frequent use of this substance (C. J. Morgan et al., 2012; C. J. A. Morgan et al., 2008). Interestingly, this was the only criterion that ketamine shared a significant relationship with, despite previous literature showing individuals who use ketamine to have increased risk for dependence and abuse (Van Amsterdam & Van Den Brink, 2022). Thus, future cross-sectional and longitudinal studies that further examine recreational ketamine use are needed to clarify ketamine’s potential for abuse and dependence.

### 4.3 Mechanisms: Classic psychedelics (LSD, mescaline)

First, LSD’s long duration of action may be a mechanism to explain the association between LSD use and significant time spent getting, using, or getting over the effects of hallucinogens. LSD is known to have one of the longest durations of action of any classic psychedelic, as the acute effects of this substance last around 12 h (Schmid et al., 2015). In addition, a recent *meta*-analysis of randomized control trials of LSD in healthy participants found that acute or subacute adverse effects (i.e., persisting panic, anxiety, etc.) could last anywhere from several hours up to twenty-four hours (Li et al., 2022). Existing research that details the rapid onset of LSD tolerance is congruent with our findings linking LSD use to decreased effectiveness with continued use. Repeated administration of LSD is known to cause individuals to rapidly develop a tolerance resulting in diminishing effects of this substance (Nichols, 2016). Further research on the frequency of LSD use among individuals with recent-onset hallucinogen use can allow for deeper understanding of the relationship between this substance and the aforementioned criterion.

Lastly, mescaline conferring increased odds of engaging in fewer important activities may be a result of its long duration of action (i.e., 10–12 h) (Dinis-Oliveira et al., 2019); furthermore, this finding is roughly in line with Stone et al. (2007), which found individuals who use mescaline to have increased risk for hallucinogen dependence. However, the aforementioned mechanism is purely speculative as little modern research has been conducted on the association between mescaline use and hallucinogen misuse. Further research is needed on the mechanisms that may underlie these potential associations.

### 4.4 Future directions

There are a few future directions warranted by our observed findings. First, as our study was cross-sectional, future longitudinal studies can assess whether our observed relationships are indeed causal. Future investigations can also examine how associations between hallucinogens and dependence and abuse change over time. Additionally, a Bayesian statistical approach will also provide us with more information about the null findings we observed for the other hallucinogens in our analyses. In the current study, we took a traditional frequentist null and alternative hypothesis testing approach. With this approach, we cannot determine whether our failure to find associations between MDMA/ecstasy, psilocybin, and misuse directly means there are no associations. A Bayesian statistical approach would allow us to conduct these inquiries and confirm our null findings. Third, based on our results, PCP was the substance most strongly associated with dependence and abuse. However, comprehensive investigations of each hallucinogen, as well as patterns of polysubstance use across different hallucinogens, will be important to truly understand the relationships between these substances and dependence and abuse behavior. Finally, future investigations can also examine how the associations between individual hallucinogens and dependence and abuse vary by demographic factors (e.g., age, race) as well.

## 5 Conclusion

This study represents a novel investigation into the link between recreational hallucinogen use and DSM-IV diagnoses of hallucinogen dependence and abuse. Overall, our study demonstrated that PCP was most strongly linked to hallucinogen dependence and abuse in a nationally-representative cross-sectional dataset. LSD, ketamine, and mescaline also shared significant associations with various hallucinogen dependence and abuse criteria as well. Future investigations can provide more clarity on potential mechanisms underlying these associations and can elucidate whether our findings are causal. In sum, we provide incremental progress towards better understanding substance misuse and the disordered behavior that might result from hallucinogens.

Data Availability Statement

The data for this study are available at the Substance Abuse & Mental Health Data Archive (SAMHDA) at the following web address: https://www.datafiles.samhsa.gov/.

## Declaration of Competing Interest

The authors declare that they have no known competing financial interests or personal relationships that could have appeared to influence the work reported in this paper.

## Data Availability

Data are publicly available online
